# Advances in the field of intranasal oxytocin research: lessons learned and future directions for clinical research

**DOI:** 10.1038/s41380-020-00864-7

**Published:** 2020-08-17

**Authors:** Daniel S. Quintana, Alexander Lischke, Sally Grace, Dirk Scheele, Yina Ma, Benjamin Becker

**Affiliations:** 1 Norwegian Centre for Mental Disorders Research (NORMENT), University of Oslo and Oslo University Hospital, Oslo, Norway; 2 Department of Psychology, University of Greifswald, Greifswald, Germany; 3 School of Psychology, Australian Catholic University, Melbourne, Australia; 4 Division of Medical Psychology, Department of Psychiatry and Psychotherapy, University Hospital Bonn, Bonn, Germany; 5 Department of Psychiatry, School of Medicine & Health Sciences, University of Oldenburg, Oldenburg, Germany; 6 State Key Laboratory of Cognitive Neuroscience and Learning, IDG/McGovern Institute for Brain Research, Beijing Key Laboratory of Brain Imaging and Connectomics, Beijing Normal University, Beijing, China; 7 The Clinical Hospital of the Chengdu Brain Science Institute, Key Laboratory for NeuroInformation, School of Life Science and Technology, Center for Information in Medicine, University of Electronic Science and Technology of China, Chengdu, China

**Keywords:** Psychology, Neuroscience

## Abstract

Reports on the modulatory role of the neuropeptide oxytocin on social cognition and behavior have steadily increased over the last two decades, stimulating considerable interest in its psychiatric application. Basic and clinical research in humans primarily employs intranasal application protocols. This approach assumes that intranasal administration increases oxytocin levels in the central nervous system via a direct nose-to-brain route, which in turn acts upon centrally-located oxytocin receptors to exert its behavioral effects. However, debates have emerged on whether intranasally administered oxytocin enters the brain via the nose-to-brain route and whether this route leads to functionally relevant increases in central oxytocin levels. In this review we outline recent advances from human and animal research that provide converging evidence for functionally relevant effects of the intranasal oxytocin administration route, suggesting that direct nose-to-brain delivery underlies the behavioral effects of oxytocin on social cognition and behavior. Moreover, advances in previously debated methodological issues, such as pre-registration, reproducibility, statistical power, interpretation of non-significant results, dosage, and sex differences are discussed and integrated with suggestions for the next steps in translating intranasal oxytocin into psychiatric applications.

## Introduction

The neuropeptide oxytocin has attracted immense research attention for its reported effects on social cognition and social behavior [1]. Motivated by studies demonstrating that the intranasal administration of the closely-related hypophyseal neuropeptide vasopressin elevates cortical arousal [2] and vasopressin levels in the cerebrospinal fluid [3], the intranasal application of oxytocin became the tool of choice for studies investigating its social cognitive effects. Intranasal oxytocin has been shown to facilitate the encoding [4, 5], consolidation [6], and recognition [7, 8] of social stimuli in various contexts. Although the precise mechanisms accounting for the effects of oxytocin on social cognition remain to be elucidated, accumulating evidence suggests that oxytocin increases overt and covert attention for social signals [8–11], which would have provided a highly adaptive function relevant for the survival of our ancestors [12]. As oxytocin does not exclusively enhance prosocial behavior [13, 14] and in certain contexts may even *enhance* anti-social behavior (e.g., envy [15], gloating [15], or aggression [16, 17]), the mediating effects of oxytocin-induced changes in social stimulus processing on social behavior are far more complex than initially thought [18]. Regardless, the effects of intranasal oxytocin are likely mediated by its modulation of neural activity in various brain regions rich in oxytocin receptors [19] that are implicated in the processing of social and affective stimuli [20, 21], such as the amygdala [22–24], insula [25–27], and striatum [28].

Owing to early reports of the beneficial effects of intranasal oxytocin on social cognition, it has been nominated as a promising pharmacological agent for the treatment of psychiatric disorders characterized by social and/or emotional dysregulations [29], such as autism [30], borderline personality disorder [31], post-traumatic stress disorder (PTSD) [32], and schizophrenia [33], in light of research using both single [34–36] and chronic administration [37] protocols. Oxytocin may also play a role in the treatment of inflammation and pain [38], weight control [39], dementia [40], and ageing-related neurocognitive decline [41]. However, the available evidence in favor of the therapeutic utility of oxytocin is currently limited. Despite the growing number of reports on the effects of intranasal oxytocin on social cognition in humans, initial failures to replicate its effects, methodological shortcomings, and a poor understanding of neurobiological mechanisms have led to critiques of the behavioral and clinical oxytocin field. These include a limited comprehension of nose-to-brain drug delivery mechanisms—specifically whether oxytocin penetrates the brain in functionally relevant amounts following intranasal application—as well as the lack of rigorous methodological features, including appropriate statistical power, replication studies, pre-registered protocols, and state-of-the-art statistical inference [42–45]. We argue that the field moved too quickly without initially accounting for essential mechanisms of action and methodological rigor, which need to be addressed before fully realizing the clinical potential of oxytocin. As the field has more recently begun to address these critical issues, now is an opportune time to provide an integrative review on the corresponding progress, to identify remaining issues, and to propose a roadmap for further improving methodological standards in the field to ultimately promote the translation of oxytocin in psychiatric applications.

## Research strategies to determine the role of oxytocin in human behavior

Congruent evidence generated by diverse methodological strategies points to the role of oxytocin as a modulator of social behavior and social cognition in humans. Observational studies have reported associations between individual variations in social behavior and social cognition with endogenous oxytocin levels [46], candidate genes involved in oxytocin signaling [47, 48], and peripheral gene expression [49]. However, general methodological issues such as the inability to control for unspecific confounders in individual difference approaches and statistically underpowered candidate gene studies [50], as well as more oxytocin-specific issues, including; (i) weak associations between endogenous peripheral and central oxytocin levels [51] (but also see [52]), (ii) immunological assay problems [53, 54], and (iii) the replicability of candidate gene associations in meta-analyses [55] hamper a clear interpretation of these findings. To overcome these limitations, experimental studies have manipulated oxytocin levels by administering exogenous oxytocin in randomized placebo-controlled double-blind designs, with meta-analytic studies suggesting that oxytocin administration improves the recognition of basic emotions in neurotypical participants [56, 57], and high-level social cognition in severe psychiatric disorders [58]. Strategies to transiently enhance oxytocin levels include intravenous [59, 60] and intranasal oxytocin administration [61, 62].

## Does intranasal oxytocin reach the brain?

**Fig. 1:**
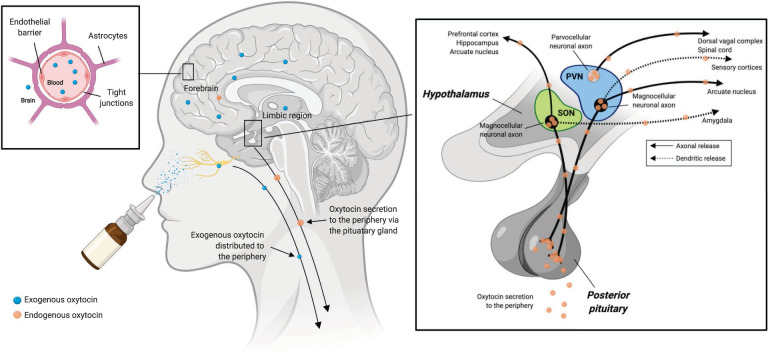
Endogenous oxytocin administration and endogenous oxytocin production. Endogenous oxytocin is primarily produced in the hypothalamus, within the supraoptic (SON) and paraventricular (PVN) nuclei (right inset). Synthesized oxytocin is stored for peripheral release in the posterior pituitary and also secreted within the brain via axonal and dendritic release mechanisms. Intranasally administered exogenous oxytocin travels both to the brain, via olfactory and trigeminal nerve fibers, and the periphery via the highly vascularized nasal cavity. Only very small amounts of peripherally circulating oxytocin are thought to cross the blood-brain barrier owing to an endothelial barrier with tight junctions (left inset), however, these amounts might still be biologically relevant. Image created with BioRender.com.

Several lines of research indicate that intranasally administered oxytocin reaches the brain. Given that only a small fraction of peripheral oxytocin passes the blood-brain barrier after intravenous administration [63], as compared to intranasal application [3, 64], intranasal administration has become the most commonly used experimental protocol for uncovering the role of central oxytocin signaling in humans (Fig. 1). Moreover, intranasal administration is a non-invasive and well-tolerated means of drug delivery which has recently gained increasing interest as an administration route for psychotropic agents, such as esketamine for treatment-resistant depression [65]. The reported number of side effects after intranasal oxytocin  administration are not significantly greater than placebo, either in children [66, 67] or adults [68]. Despite the appeal and popularity of the intranasal oxytocin administration approach, this method has been criticized on a number of grounds, including doubts whether oxytocin penetrates into the central nervous system and whether the low amounts of oxytocin that reach the central nervous system can actually lead to functionally relevant effects [43, 69], the suitability of commonly used pump-actuated devices to deliver oxytocin to the brain [70], and drug delivery issues associated with the labyrinth-like nasal cavity [71]. In the following sections, we will provide an overview of recent direct evidence that administration of exogenous oxytocin can reach the brain in biologically and functionally relevant amounts.

### Intranasal vs. intravenous administration

A comparison of the effects of intranasal and intravenous oxytocin administration demonstrates that intranasal oxytocin travels to the brain directly and that social-cognitive effects of intranasal oxytocin are not primarily due to effects on peripheral oxytocin receptors. Specifically, Quintana et al. demonstrated that despite comparable peripheral oxytocin levels after intravenous and intranasal administration (administered using a double-dummy design), social cognitive [72] and neural effects [73] were only observed after intranasal administration. This indicates that intranasally administered oxytocin is not likely to reach the brain by crossing the blood-brain barrier via the circulatory system, but rather, through direct transport via olfactory and trigeminal nerve fibers that innervate the nasal cavity (Fig. 1). These results also suggest that increasing peripheral levels of oxytocin via intravenous administration does not appear to influence social cognition and neural activity via activation of peripheral oxytocin receptors, at least with a 1 IU intravenous dose. Similarly, nonhuman primate data suggests that intranasal, but not intravenous, oxytocin administration increases central endogenous oxytocin release [74]. In fact, it is likely that the effects observed in human studies, which typically assess cognitive performance and neurobiological measures 40–60 min after administration are due to increases in endogenous oxytocin production, as the half-life of exogenous oxytocin in the cerebrospinal fluid (CSF) is estimated to be about 19 min [75]. Martins et al. [76] reported that both intranasal and intravenous administration reduces amygdala and anterior cingulate cortex perfusion, suggesting that at least for these brain regions, intranasal oxytocin may not necessarily use a privileged nose-to-brain route. However, there were unique oxytocin-induced increases in brain perfusion in other brain regions after intranasal administration, which also differed according to the intranasal device used (i.e., a standard pump-actuated device vs. a nebulizer). Of note, the intravenous dose administered in this study [76] was 10-fold higher compared to Quintana et al. [73]. Perhaps, once an intravenous dose reaches a certain threshold it will cross the blood-brain barrier and influence brain activity in regions with high oxytocin receptor density, such as the amygdala [19], but not other brain regions with lower densities [76]. This is consistent with recent work using primate [74] and rodent [77] models. However, higher peripheral doses have the potential to increase the risk of unanticipated effects via peripheral oxytocin receptor activation or cross-reaction with vasopressin receptors [43].

### Functionally relevant increases in the CSF after intranasal administration

Functionally relevant increases in oxytocin in CSF after intranasal administration demonstrates this approach can raise central levels for activation of oxytocin receptors in the brain, which can influence cognition and behavior. Peripheral concentrations of oxytocin are often measured after intranasal oxytocin administration to indicate an increase in central levels of oxytocin. Although a recent meta-analysis suggests that central and peripheral levels of oxytocin are strongly and significantly correlated after intranasal administration [51], peripheral concentrations still only provide an indirect estimate of central oxytocin concentrations [51]. As the calculation of peripheral oxytocin levels has been questioned on methodological grounds [78] (but see [53]), the determination of central oxytocin levels in the CSF has become an important tool to assess the effects of intranasal oxytocin administration. By now, several animal [74, 79–82] and human [83] studies have demonstrated that intranasally administered oxytocin increases oxytocin levels in CSF.

Some of the clearest evidence that intranasal oxytocin was effective to penetrate into the brain in rat and primate models was reported in recent studies [74, 84]. Lee et al. [74] measured CSF concentrations of oxytocin after intranasal administration of labeled (d5-deuterated) oxytocin and provided direct evidence for CSF penetrance of intranasal oxytocin administered to nonhuman primates. A follow-up study using a similar approach demonstrated that labeled oxytocin delivered intranasally, but not intravenously, reaches several brain regions innervated by trigeminal and olfactory nerve fibers [85]. Another recent investigation [84] systematically examined the pharmacokinetic properties and brain distribution of oxytocin after intranasal application in rats. This study evaluated the disposition, nasal absorption, and bioavailability of oxytocin after nasal administration, and showed evidence that the nasal bioavailability of oxytocin was approximately 2%, and more than 95% of oxytocin in the brain was directly transported from the nasal cavity. Of particular interest, a recent study by Smith et al. [82] demonstrated that increases in central concentrations are observed in oxytocin knockout mice, demonstrating that the source of observed increases is likely to be exogenous oxytocin. Moreover, by examining extracellular fluid from specific brain structures, Smith et al. [82] were able to show that intranasally administered oxytocin permeates target regions of the brain, such as the amygdala and the hippocampus, to a degree considered to be functionally relevant [86]. Using oxytocin-induced CSF change as an indicator, a study by Rault [87] measured cerebrospinal fluid samples before and after intranasal administration of 50 μg oxytocin in pigs (close to the human equivalent dose of 24 IU, considering body surface area, pharmacokinetics, and physiological time differences among species [88]), showing that 50 ng reached the CSF. Although only a small proportion accessed the brain, the common dose of 24 IU providing 50 ng reaching the CSF influences neural activity and is considered a supra-physiological dose given the commonly reported baseline endogenous CSF oxytocin concentrations in humans [83, 89]. Altogether, accumulating research points to intranasal oxytocin increasing central levels of oxytocin, to a functionally relevant degree.

### Oxytocin radiotracers

The development of oxytocin receptor-specific radioligands would provide the means to directly trace the binding sites of exogenous oxytocin administration [90, 91]. However, the poor selectivity and/or insufficient potency for the oxytocin receptor [92] and limited brain penetration [92] have hindered the search for highly-specific oxytocin radioligands. Indeed, there are currently no ligands that can be applied in human research. More recently, the development of a novel Al^18^F oxytocin receptor tracer with high potency and selectivity [93] identified higher binding in the oxytocin receptor-rich olfactory bulb region after intranasal compared to intravenous administration in rats, which is consistent with direct nose-to-brain transfer. While this is promising, research is needed in humans considering differences in oxytocin receptor locations between species [94].

## What is the most efficacious dose and timing of intranasal oxytocin?

Dose-ranging research is a crucial and underrecognized step for translational oxytocin research [29]. Without understanding the dose-response pattern, non-significant effects may simply be due to an incorrect dosage, rather than a lack of efficacy. Higher doses may also lead to the occupation of vasopressin receptors, which could contribute to the observed behavioral effects, but also side-effects [95]. To better understand the dose-response, Galbusera et al. [96] mapped the brain-wide functional substrates engaged by acute intranasal oxytocin in mice and demonstrated rapid and sustained activation in areas with high oxytocin receptor density, including key regions engaged in social and emotional behavior. Importantly, they showed that the different regional dynamics produced by intranasal oxytocin was dose-invariant between the low dose (i.e., 0.33 μg/mouse) and a dose that was four times higher (i.e., 1.33 μg/mouse). The low oxytocin dose equals 52.8 μg (i.e., 26.4 IU) in a human individual (transformed by weight; [30 g/mouse; 60 kg/human [88]). It is also worth noting that a common finding in recent oxytocin studies is that intranasal oxytocin does not produce a linear dose-response curve [97–99]. Although the exact mechanism is unknown, it has been speculated that the non-linearity of oxytocin dose-response is due to coupling with different G proteins or binding to the *AVPR1A* receptor when high doses flood available oxytocin receptors [100]. Of interest, Guoynes et al. [98] examined the effect of intranasal oxytocin on the central receptor binding and immunoreactive protein for oxytocin and detected significant changes in the prairie voles receiving a dose similar to the equivalent in human studies but not for lower or higher doses.

Human intranasal oxytocin studies primarily employ dosages between 20 and 48 IU [20, 21], with the majority using a 24 IU dose, and examine behavioral and neural effects in timeframes of 20–90 min after drug administration. Studies in children often adjust dosages by bodyweight (e.g., [101]), but no clear guidelines currently exist on which doses should be used when taking this approach. Four studies have systematically examined intranasal oxytocin brain responses at varying doses in humans. Quintana et al. [73] reported that 8 IU—but not 24 IU—is the most efficacious intranasal oxytocin dose to elicit an amygdala response to emotional faces; whereas, Spengler et al. [102] identified that a time window between 45 and 70 min and a dose of 24 IU—not 12 or 48 IU—elicited the most robust decreases in amygdala response to emotional faces, to a greatest extent in those with high (versus low) autistic traits. It is possible that the novel breath-powered device used by Quintana et al. [73] allowed for a greater dose of oxytocin to enter the nasal cavity at 8 IU and therefore elicited similar effects centrally as a standard 24 IU nasal spray used by Spengler et al. [102]. Nonetheless, the different dosage effects indirectly confirm dose–response effects on central processing. Pharmacodynamic studies are also in line with the nose-to-brain temporal pattern. Lieberz et al. [103] administered 6, 12, and 24 IU to healthy women and observed an increase in amygdala reactivity across doses indicating that sex-specific treatment effects are not only a byproduct of a shifted dose-dependent target engagement. Paloyelis et al. [104] measured the availability of intranasal oxytocin to brain tissues in human participants using arterial spin labeling (ASL) to quantify in vivo intranasal oxytocin-induced changes in resting regional cerebral blood flow (rCBF), which reflects changes in neuronal activity while controlling for secondary vascular effects. The study demonstrated intranasal oxytocin-induced changes in regions expected to express oxytocin receptors [19], including limbic and midbrain/brainstem regions such as the amygdala, hippocampus, caudate nucleus, ventral striatum and pallidum, anterior and middle cingulate, inferior frontal gyrus, anterior insula, and superior temporal gyrus, and these changes were sustained over the entire observation interval of 78 min. The pharmacokinetic profile of oxytocin on regional brain activation additionally seems to vary depending on the brain region [76], suggesting that a one-fits-all for determining optimal post-dose timing might be too simplistic. In sum, emerging evidence suggests differential effects of oxytocin dose on brain responses and behaviors, with the caveat that varying administration devices [73, 76], social cognitive domains examined [105], sex [103], timing [76], or the mental state of the individual [102] may differentially affect responses. We advocate that future studies are needed to systematically evaluate the dose-response curve and dose-time of intranasal oxytocin in different populations and contexts to exert the desired behavioral and neural effects. While previous studies investigating the effects of intranasal oxytocin on amygdala reactivity were not moderated by weight [102, 103], the impact of body weight on the most efficacious intranasal oxytocin dose, particularly in children, should be further investigated.

## Future directions for clinical research using intranasal oxytocin

Concerns over questionable research practices have led to skepticism regarding published effects in oxytocin research [42, 106], however, there are several methodological practices that can increase the credibility of intranasal oxytocin research (Table 1). First, to reduce bias researchers should pre-register their hypothesis-driven studies including their hypotheses, methodological details, and statistical power calculations prior to data collection. While preregistration is the norm in clinical research, conventional registrations typically lack sufficiently detailed analysis plans, which leave considerable room for analytical flexibility as there are millions of ways to analyze a typical dataset [107], which increases the risk of false positives. An alternative option to standard trial registration is to use the Registered Reports format [e.g., 108], in which articles are granted in-principle acceptance *before* data is collected, based on strength of their rationale and methodological plan [109]. As well as helping ensure that researchers follow their pre-specified analysis plan, Registered Reports also reduce publication bias as results are published regardless of statistical significance. While the pre-registration of detailed analysis plans for neuroimaging studies is less straightforward, recent recommendations aim at increasing the transparency and reproducibility of neuroimaging research [110] and tools have recently emerged to assist this process, such as pre-registration templates [111] and fMRI data simulation [112]. Second, future clinical studies should aim at recruiting both male and female participants to improve generalizability. Despite evidence for sex-specific roles of oxytocin [23, 24, 34, 103, 113] most human research has been conducted in male samples or did not account in their analysis for potential sex-differences [114, 115]. If resources are too limited to include both sexes, there should be an increased focus in testing female participants and understanding which results from male populations replicate in females, where possible. Third, direct replications by independent labs are required, which should also investigate the impact of different intranasal oxytocin dosages in psychiatric populations. Despite the initial lack of replication in the domains of mind reading and trust [14, 106, 108, 116], more recent studies suggest partly replicable effects in the domains of emotional empathy and self-referential processing [105, 117]. Fourth, other researchers should be able to reproduce the results of reported studies using open data and analysis scripts. Of course, participant privacy is paramount, however, many research participants are supportive of open participant data [118, 119]. Regardless, the use of synthetic data sets [120], which share the same statistical characteristics as the original dataset while reducing the disclosure risk to essentially nil can help sidestep privacy concerns (for examples using data from an oxytocin administration study, see [121]). Fifth, the reporting of null results needs to increase along with the accurate interpretation of such results. While scientific journals can be reluctant to publish non-significant results [122], this attitude is certainly shifting (e.g., [106]). To better understand non-significant results, researchers should adopt emerging statistical methods such as Bayesian hypothesis testing [123, 124] or frequentist equivalence testing [125] on appropriately powered samples to assess the evidence for meaningful nonsignificant results [126].

**Table 1:** Key issues and potential solutions for improving methodological standards for the field of human intranasal oxytocin research.

Issue	How this issue hinders translation	Potential solutions
Researcher bias in analytic decisions	Biased studies are less likely to replicate	Detailed pre-registrations or Registered Reports
Underrepresentation of females	Most research is only applicable to males	More studies with female populations
Few studies have been replicated	It is unclear which results are robust	More direct replication studies
Open data and analysis scripts	Reported results are harder to verify	Obtain consent to share anonymized data or create synthetic data
Interpreting non-significant results	It is difficult to falsify hypotheses	Equivalence testing and Bayesian hypothesis testing
Domain-specific theories	Difficult to interpret results in new domains	Development of theories that are applicable across broad domains
Low statistical power	Studies are less likely to replicate	Within-participant designs and one-sided tests (when warranted)
Generalizability of results	Results cannot be generalized to other populations	Identify which findings from neurotypical populations replicate in clinical groups

**Fig. 2:**
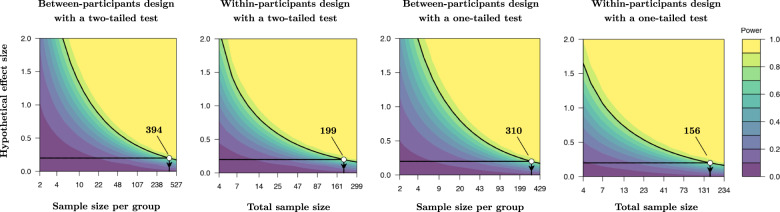
Minor changes in study design can dramatically reduce the resources required to achieve desired statistical power for intranasal oxytocin studies. Four power contour plots are presented for different study designs, which all detect an effect size of *δ* = 0.2 with a Type I error rate of α = 0.05 and 80% power. Power contour plots demonstrate how the sensitivity of a test changes with the hypothetical effect size and sample size. The most efficient use of resources from these examples is a within-participants design using a one-tailed test. With this research design, a sample size of 156 participants is required to reliably detect (with a probability greater than 80%) an effect size of δ ≥ 0.2. In other words, this design requires the resources to support 312 laboratory visits in total. In comparison, the a between-participants design with a two-tailed test with the same parameters requires 788 laboratory visits in total.

Low statistical power is a well-known issue for intranasal oxytocin research [42]. There are two relatively straightforward approaches that can increase the power of intranasal oxytocin studies. Compared to a between-participants study with the same number of participants, a within-participant design provides superior power ([127] Fig. 2). However, potential learning and memory effects may prohibit repeated testing in a cross-over treatment design. Pre-registered one-tailed *p*-value thresholds, which are recommended for hypothesis-driven studies [128], can also increase tatistical power without using any additional resources (Fig. 2). For example, detecting an effect size of *δ* = 0.2 (with a Type I error rate of α = 0.05 and 80% power) using a between-participants design and a two-tailed test requires a sample size of 394 *per group* (i.e., the recruitment of 788 participants). However, using the same parameters with a between-participants design and a one-tailed test only requires the recruitment of 156 participants. In terms of meta-analyses, raw data studies can be combined for individual participant data meta-analysis, which facilitates more powerful moderation analysis (e.g., sex, psychiatric illness diagnosis) than conventional meta-analysis.

In parallel with recent appeals to increase reproducibility by improving methodology, some have called for a greater focus on theory development to improve reproducibility [129–131]. Broadly speaking, these arguments suggest that the source of reproducibility issues in the behavioral sciences is not exclusively methodological in nature, but rather, researchers are asking the wrong questions in the first place. By reframing poor reproducibility of oxytocin research in this light, failures to replicate early findings that oxytocin increases trusting behaviors could also be explained by the mistaken theoretical belief that oxytocin increases prosocial behaviors. Of course, both theoretical and methodological failures contribute to poor reproducibility. Thus, in the current push to improve methodology, theory should not be left by the wayside and more effort should be devoted to developing theories with clear and falsifiable predictions that are relevant across domains. One such theoretical approach that is gaining popularity in the medical sciences is an evolutionary framework (e.g., [132]), as such theories can predict and explain results across contexts [129]. Reflecting the generalizability this framework, two recent testable oxytocin theories have emerged that embrace an evolutionary framework, a life history theory that suggests oxytocin mediates resource allocation [133] and an allostatic theory that proposes that oxytocin helps maintain stability in changing environments [12]. Of note, both these theories can account for the effects of oxytocin on non-social cognition and behavior [134].

The previous methodological recommendations largely pertain to issues in the biobehavioral science in general. We also provide a few additional suggestions specifically relevant to intranasal oxytocin research. First, future research should demonstrate the similarities and differences between the effects of oxytocin on cognition and physiology in healthy and clinical populations. In other words, are findings on the effect of intranasal oxytocin in neurotypical populations generalizable to clinical populations? Most claims regarding the therapeutic utility of oxytocin have been based on proof-of-principle studies that investigated the effects of oxytocin on social cognitive functions in healthy individuals [135]. A series of meta-analyses on the effects of intranasal oxytocin on the interpretation of emotions demonstrated similar effect sizes between clinical and neurotypical populations [57]. However, the confidence intervals tended to be considerably wider for the clinical population meta-analyses, which probably reflects the heterogeneity of response between different psychiatric disorders. In terms of neural activity, a meta-analysis of 66 fMRI studies concluded that intranasal oxytocin administration decreases amygdala activity in both neurotypical and psychiatric populations [20]. However, this meta-analysis also revealed that intranasal oxytocin seems to have divergent effects between neurotypical and psychiatric populations on neural activity in other brain regions (e.g., superior temporal, dorsal anterior cingulate). Moreover, accumulating evidence suggests that the behavioral and neural effects of oxytocin in healthy individuals vary as a function of psychopathology relevant dimensions, like, for example, alexithymia [136], autism [137, 138], depression [139–141], anxiety [142, 143], or trauma-exposure [144, 145]. Although a pathology-dependent variation in oxytocin’s effects may emphasize the clinical potential of oxytocin, it may also indicate that oxytocin may have divergent effects in psychiatric and healthy populations [34]. To this end, it will be important to demonstrate that the effects of oxytocin in neurotypical populations translate to clinical populations, and which disorders in particular can most benefit from intranasal oxytocin treatment.

Second, it is unclear whether chronic administration of oxytocin will emerge as a long-term treatment for patients [146], or if short-term adjunctive treatment with oxytocin might be more appropriate. Given the manifold effects of oxytocin on social cognitive functions, it seems conceivable that oxytocin is capable to augment therapeutic interventions in various ways. For instance, during patient-therapist interactions, oxytocin may enhance patients’ attention for the therapists’ verbal and non-verbal signals [8, 147], the intention to follow instructions [147, 148], and the willingness to build a working alliance with the therapist [149].

## Summary

Converging translational evidence from recent studies suggests that oxytocin reaches the brain in biological and behavioral relevant amounts following intranasal administration. More recently, the field of oxytocin research has increasingly adopted more rigorous methodological practices, including replication designs in comparably large samples [108, 117], increasing publication of null effects [150, 151], and pre-registration of basic research and clinical intranasal oxytocin studies. Despite lacking a complete picture of the biological mechanisms of the oxytocin signaling pathways in humans and the precise role of oxytocin in human social behavior and social cognition, initial studies in patient populations suggest a potential use as novel therapeutic strategy in psychiatric disorders characterized by social dysfunction [30, 31, 33]. Growing clinical interest is not only reflected by the increasing number of studies but also the unsupervised off-label use of oxytocin for the treatment of psychiatric disorders (e.g., [152]). The urgent need for a therapeutic to treat social dysfunction and lay interest in this treatment should not come at the expense of careful, rigorous, and replicable science. Recent critiques of human intranasal oxytocin have been a much-needed wake-up call for behavioral oxytocin research. While there are certainly some mechanistic gaps that need to be filled and the need for broader adoption of reproducible scientific practices, the intranasal administration of oxytocin remains a worthwhile approach to better understand the neurobiology of our thoughts, feelings, and behaviors.

## References

[1] Kenkel WM (2019). Corpus colossal: a bibliometric analysis of neuroscience abstracts and impact factor. Front Integr Neurosci..

[2] Fehm-Wolfsdorf G, Bachholz G, Born J, Voigt K, Fehm HL (1988). Vasopressin but not oxytocin enhances cortical arousal: an integrative hypothesis on behavioral effects of neurohypophyseal hormones. Psychopharmacol.

[3] Born J, Lange T, Kern W, McGregor GP, Bickel U, Fehm HL (2002). Sniffing neuropeptides: a transnasal approach to the human brain. Nat Neurosci..

[4] Guastella AJ, Mitchell PB, Mathews F (2008). Oxytocin enhances the encoding of positive social memories in humans. Biol Psychiatry..

[5] Rimmele U, Hediger K, Heinrichs M, Klaver P (2009). Oxytocin makes a face in memory familiar. J Neurosci..

[6] Savaskan E, Ehrhardt R, Schulz A, Walter M, Schächinger H (2008). Post-learning intranasal oxytocin modulates human memory for facial identity. Psychoneuroendocrinology..

[7] Fischer-Shofty M, Shamay-Tsoory SG, Harari H, Levkovitz Y (2010). The effect of intranasal administration of oxytocin on fear recognition. Neuropsychologia..

[8] Lischke A, Berger C, Prehn K, Heinrichs M, Herpertz SC, Domes G (2012). Intranasal oxytocin enhances emotion recognition from dynamic facial expressions and leaves eye-gaze unaffected. Psychoneuroendocrinology..

[9] Schulze L, Lischke A, Greif J, Herpertz SC, Heinrichs M, Domes G (2011). Oxytocin increases recognition of masked emotional faces. Psychoneuroendocrinology..

[10] Domes G, Steiner A, Porges SW, Heinrichs M (2013). Oxytocin differentially modulates eye gaze to naturalistic social signals of happiness and anger. Psychoneuroendocrinology..

[11] Prehn K, Kazzer P, Lischke A, Heinrichs M, Herpertz SC, Domes G (2013). Effects of intranasal oxytocin on pupil dilation indicate increased salience of socioaffective stimuli. Psychophysiology..

[12] Quintana DS, Guastella AJ (2020). An allostatic theory of oxytocin. Trends Cogn Sci.

[13] Klackl J, Pfundmair M, Agroskin D, Jonas E (2013). Who is to blame? Oxytocin promotes nonpersonalistic attributions in response to a trust betrayal. Biol Psychol..

[14] Lane A, Mikolajczak M, Treinen E, Samson D, Corneille O, Timary Pde (2015). Failed replication of oxytocin effects on trust: the envelope task case. PLOS ONE..

[15] Shamay-Tsoory SG, Fischer M, Dvash J, Harari H, Perach-Bloom N, Levkovitz Y (2009). Intranasal administration of oxytocin increases envy and schadenfreude (gloating). Biol Psychiatry..

[16] Zhang H, Gross J, De Dreu C, Ma Y (2019). Oxytocin promotes coordinated out-group attack during intergroup conflict in humans. ELife..

[17] Ne’eman R, Perach-Barzilay N, Fischer-Shofty M, Atias A, Shamay-Tsoory SG (2016). Intranasal administration of oxytocin increases human aggressive behavior. Horm Behav..

[18] Bartz JA, Zaki J, Bolger N, Ochsner KN (2011). Social effects of oxytocin in humans: context and person matter. Trends Cogn Sci.

[19] Quintana DS, Rokicki J, van der Meer D, Alnæs D, Kaufmann T, Córdova-Palomera A (2019). Oxytocin pathway gene networks in the human brain. Nat Commun..

[20] Wang D, Yan X, Li M, Ma Y (2017). Neural substrates underlying the effects of oxytocin: a quantitative meta-analysis of pharmaco-imaging studies. Soc Cogn Affect Neurosci.

[21] Grace SA, Rossell SL, Heinrichs M, Kordsachia C, Labuschagne I (2018). Oxytocin and brain activity in humans: a systematic review and coordinate-based meta-analysis of functional MRI studies. Psychoneuroendocrinology..

[22] Domes G, Heinrichs M, Gläscher J, Büchel C, Braus DF, Herpertz SC (2007). Oxytocin attenuates amygdala responses to emotional faces regardless of valence. Biol Psychiatry..

[23] Domes G, Lischke A, Berger C, Grossmann A, Hauenstein K, Heinrichs M (2010). Effects of intranasal oxytocin on emotional face processing in women. Psychoneuroendocrinology..

[24] Lischke A, Gamer M, Berger C, Grossmann A, Hauenstein K, Heinrichs M (2012). Oxytocin increases amygdala reactivity to threatening scenes in females. Psychoneuroendocrinology..

[25] Striepens N, Scheele D, Kendrick KM, Becker B, Schäfer L, Schwalba K (2012). Oxytocin facilitates protective responses to aversive social stimuli in males. Proc Natl Acad Sci.

[26] Yao S, Becker B, Zhao W, Zhao Z, Kou J, Ma X (2018). Oxytocin modulates attention switching between interoceptive signals and external social cues. Neuropsychopharmacology..

[27] Yao S, Zhao W, Geng Y, Chen Y, Zhao Z, Ma X (2018). Oxytocin facilitates approach behavior to positive social stimuli via decreasing anterior insula activity. Int J Neuropsychopharmacol.

[28] Scheele D, Wille A, Kendrick KM, Stoffel-Wagner B, Becker B, Güntürkün O (2013). Oxytocin enhances brain reward system responses in men viewing the face of their female partner. Proc Natl Acad Sci.

[29] Insel TR (2016). Translating oxytocin neuroscience to the clinic: a national institute of mental health perspective. Biol Psychiatry.

[30] Guastella AJ, Hickie IB (2016). Oxytocin treatment, circuitry and autism: a critical review of the literature placing oxytocin into the autism context. Biol Psychiatry.

[31] Herpertz SC, Bertsch K (2015). A new perspective on the pathophysiology of borderline personality disorder: a model of the role of oxytocin. Am J Psychiatry.

[32] van Zuiden M, Frijling JL, Nawijn L, Koch SB, Goslings JC, Luitse JS (2017). Intranasal oxytocin to prevent posttraumatic stress disorder symptoms: a randomized controlled trial in emergency department patients. Biol Psychiatry..

[33] Feifel D, Shilling PD, MacDonald K (2016). A review of oxytocin’s effects on the positive, negative, and cognitive domains of schizophrenia. Biol Psychiatry..

[34] Lischke A, Herpertz SC, Berger C, Domes G, Gamer M (2017). Divergent effects of oxytocin on (para-)limbic reactivity to emotional and neutral scenes in females with and without borderline personality disorder. Soc Cogn Affect Neurosci.

[35] Labuschagne I, Phan KL, Wood A, Angstadt M, Chua P, Heinrichs M (2010). Oxytocin attenuates amygdala reactivity to fear in generalized social anxiety disorder. Neuropsychopharmacology..

[36] Domes G, Heinrichs M, Kumbier E, Grossmann A, Hauenstein K, Herpertz SC (2013). Effects of intranasal oxytocin on the neural basis of face processing in autism spectrum disorder. Biol Psychiatry..

[37] Yatawara CJ, Einfeld SL, Hickie IB, Davenport TA, Guastella AJ (2016). The effect of oxytocin nasal spray on social interaction deficits observed in young children with autism: a randomized clinical crossover trial. Mol Psychiatry..

[38] Boll S, Almeida de Minas AC, Raftogianni A, Herpertz SC, Grinevich V (2018). Oxytocin and pain perception: from animal models to human research. Neuroscience..

[39] Plessow F, Marengi DA, Perry SK, Felicione JM, Franklin R, Holmes TM (2018). Effects of intranasal oxytocin on the blood oxygenation level-dependent signal in food motivation and cognitive control pathways in overweight and obese men. Neuropsychopharmacology..

[40] Jesso S, Morlog D, Ross S, Pell MD, Pasternak SH, Mitchell DG, et al. The effects of oxytocin on social cognition and behaviour in frontotemporal dementia. Brain. 2011;134:2493–501. 10.1093/brain/awr171 21859765

[41] Huffmeijer R, Huffmeijer R, IJzendoorn MH, van, Bakermans-Kranenburg MJ (2013). Ageing and oxytocin: a call for extending human oxytocin research to ageing populations – a mini-review. Gerontology..

[42] Walum H, Waldman ID, Young LJ (2016). Statistical and methodological considerations for the interpretation of intranasal oxytocin studies. Biol Psychiatry..

[43] Churchland PS, Winkielman P (2012). Modulating social behavior with oxytocin: how does it work? What does it mean?. Horm Behav..

[44] Quintana DS, Woolley JD (2016). Intranasal oxytocin mechanisms can be better understood but its effects on social cognition and behavior are not to be sniffed at. Biol Psychiatry..

[45] Calin-Jageman RJ, Cumming G (2019). The new statistics for better science: ask how much, how uncertain, and what else is known. Am Stat..

[46] Parker KJ, Garner JP, Libove RA, Hyde SA, Hornbeak KB, Carson DS (2014). Plasma oxytocin concentrations and OXTR polymorphisms predict social impairments in children with and without autism spectrum disorder. Proc Natl Acad Sci.

[47] Ebstein RP, Knafo A, Mankuta D, Chew SH, San Lai P (2012). The contributions of oxytocin and vasopressin pathway genes to human behavior. Horm Behav.

[48] Kranz TM, Kopp M, Waltes R, Sachse M, Duketis E, Jarczok TA (2016). Meta-analysis and association of two common polymorphisms of the human oxytocin receptor gene in autism spectrum disorder. Autism Res.

[49] Chong A, Becker B, Angeles DC, Matos MG, Yue X, Lai PS, et al. The creative mind: blending oxytocinergic, dopaminergic and personality. BioRxiv. 2019:700807, https://www.biorxiv.org/content/10.1101/700807v1.

[50] Border R, Johnson EC, Evans LM, Smolen A, Berley N, Sullivan PF (2019). No support for historical candidate gene or candidate gene-by-interaction hypotheses for major depression across multiple large samples. Am J Psychiatry.

[51] Valstad M, Alvares GA, Egknud M, Matziorinis AM, Andreassen OA, Westlye LT (2017). The correlation between central and peripheral oxytocin concentrations: a systematic review and meta-analysis. Neurosci Biobehav Rev.

[52] Lefevre A, Mottolese R, Dirheimer M, Mottolese C, Duhamel J-R, Sirigu A (2017). A comparison of methods to measure central and peripheral oxytocin concentrations in human and non-human primates. Sci Rep..

[53] MacLean EL, Wilson SR, Martin WL, Davis JM, Nazarloo HP, Carter CS (2019). Challenges for measuring oxytocin: the blind men and the elephant?. Psychoneuroendocrinology..

[54] Szeto A, McCabe PM, Nation DA, Tabak BA, Rossetti MA, McCullough ME (2011). Evaluation of enzyme immunoassay and radioimmunoassay methods for the measurement of plasma oxytocin. Psychosom Med.

[55] Bakermans-Kranenburg MJ, van Ijzendoorn MH (2014). A sociability gene? Meta-analysis of oxytocin receptor genotype effects in humans. Psychiatr Genet.

[56] Shahrestani S, Kemp AH, Guastella AJ (2013). The impact of a single administration of intranasal oxytocin on the recognition of basic emotions in humans: a meta-analysis. Neuropsychopharmacology..

[57] Leppanen J, Ng KW, Tchanturia K, Treasure J (2017). Meta-analysis of the effects of intranasal oxytocin on interpretation and expression of emotions. Neurosci Biobehav Rev.

[58] Bürkner P-C, Williams DR, Simmons TC, Woolley JD (2017). Intranasal oxytocin may improve high-level social cognition in schizophrenia, but not social cognition or neurocognition in general: a multilevel bayesian meta-analysis. Schizophr Bull..

[59] Hollander E, Novotny S, Hanratty M, Yaffe R, DeCaria CM, Aronowitz BR (2003). Oxytocin infusion reduces repetitive behaviors in adults with autistic and asperger’s disorders. Neuropsychopharmacology..

[60] Hollander E, Bartz J, Chaplin W, Phillips A, Sumner J, Soorya L (2007). Oxytocin increases retention of social cognition in autism. Biol Psychiatry.

[61] Heinrichs M, Baumgartner T, Kirschbaum C, Ehlert U (2003). Social support and oxytocin interact to suppress cortisol and subjective responses to psychosocial stress. Biol Psychiatry..

[62] Kirsch P, Esslinger C, Chen Q, Mier D, Lis S, Siddhanti S (2005). Oxytocin modulates neural circuitry for social cognition and fear in humans. J Neurosci..

[63] Kang Y-S, Park J-H (2000). Brain uptake and the analgesic effect of oxytocin— its usefulness as an analgesic agent. Arch Pharm Res.

[64] Chang SW, Barter JW, Ebitz RB, Watson KK, Platt ML (2012). Inhaled oxytocin amplifies both vicarious reinforcement and self reinforcement in rhesus macaques (Macaca mulatta). Proc Natl Acad Sci.

[65] Popova V, Daly EJ, Trivedi M, Cooper K, Lane R, Lim P (2019). Efficacy and safety of flexibly dosed esketamine nasal spray combined with a newly initiated oral antidepressant in treatment-resistant depression: a randomized double-blind active-controlled study. Am J Psychiatry.

[66] DeMayo MM, Song YJC, Hickie IB, Guastella AJ (2017). A review of the safety, efficacy and mechanisms of delivery of nasal oxytocin in children: therapeutic potential for autism and prader-willi syndrome, and recommendations for future research. pediatr. Drugs..

[67] Verhees MWFT, Houben J, Ceulemans E, Bakermans-Kranenburg MJ, van IJzendoorn MH, Bosmans G (2018). No side-effects of single intranasal oxytocin administration in middle childhood. Psychopharmacology.

[68] MacDonald E, Dadds MR, Brennan JL, Williams K, Levy F, Cauchi AJ (2011). A review of safety, side-effects and subjective reactions to intranasal oxytocin in human research. Psychoneuroendocrinology..

[69] Leng G, Ludwig M (2016). Intranasal oxytocin: myths and delusions. Biol Psychiatry.

[70] Quintana DS, Guastella AJ, Westlye LT, Andreassen OA (2016). The promise and pitfalls of intranasally administering psychopharmacological agents for the treatment of psychiatric disorders. Mol Psychiatry..

[71] Quintana DS, Smerud KT, Andreassen OA, Djupesland PG (2018). Evidence for intranasal oxytocin delivery to the brain: recent advances and future perspectives. Ther Deliv..

[72] Quintana DS, Westlye LT, Rustan ØG, Tesli N, Poppy CL, Smevik H (2015). Low dose oxytocin delivered intranasally with Breath Powered device affects social-cognitive behavior: a randomized 4-way crossover trial with nasal cavity dimension assessment. Transl Psychiatry..

[73] Quintana DS, Westlye LT, Alnæs D, Rustan Ø, Kaufmann T, Smerud K (2016). Low dose intranasal oxytocin delivered with Breath Powered device dampens amygdala response to emotional stimuli: a peripheral effect-controlled within-subjects randomized dose-response fMRI trial. Psychoneuroendocrinology..

[74] Lee MR, Scheidweiler KB, Diao XX, Akhlaghi F, Cummins A, Huestis MA (2018). Oxytocin by intranasal and intravenous routes reaches the cerebrospinal fluid in rhesus macaques: determination using a novel oxytocin assay. Mol Psychiatry.

[75] Mens WB, Witter A, Van Wimersma, Greidanus TB (1983). Penetration of neurohypophyseal hormones from plasma into cerebrospinal fluid (CSF): half-times of disappearance of these neuropeptides from CSF. Brain Res..

[76] Martins DA, Mazibuko N, Zelaya F, Vasilakopoulou S, Loveridge J, Oates A (2020). Effects of route of administration on oxytocin-induced changes in regional cerebral blood flow in humans. Nat Commun.

[77] Yamamoto Y, Liang M, Munesue S, Deguchi K, Harashima A, Furuhara K (2019). Vascular RAGE transports oxytocin into the brain to elicit its maternal bonding behaviour in mice. Commun. Biol.

[78] McCullough ME, Churchland PS, Mendez AJ (2013). Problems with measuring peripheral oxytocin: can the data on oxytocin and human behavior be trusted?. Neurosci Biobehav Rev.

[79] Neumann ID, Maloumby R, Beiderbeck DI, Lukas M, Landgraf R (2013). Increased brain and plasma oxytocin after nasal and peripheral administration in rats and mice. Psychoneuroendocrinology..

[80] Modi ME, Connor-Stroud F, Landgraf R, Young LJ, Parr LA (2014). Aerosolized oxytocin increases cerebrospinal fluid oxytocin in rhesus macaques. Psychoneuroendocrinology..

[81] Freeman SM, Samineni S, Allen PC, Stockinger D, Bales KL, Hwa GGC (2016). Plasma and CSF oxytocin levels after intranasal and intravenous oxytocin in awake macaques. Psychoneuroendocrinology..

[82] Smith AS, Korgan AC, Young WS (2019). Oxytocin delivered nasally or intraperitoneally reaches the brain and plasma of normal and oxytocin knockout mice. Pharm Res..

[83] Striepens N, Kendrick KM, Hanking V, Landgraf R, Wüllner U, Maier W (2013). Elevated cerebrospinal fluid and blood concentrations of oxytocin following its intranasal administration in humans. Sci Rep..

[84] Tanaka A, Furubayashi T, Arai M, Inoue D, Kimura S, Kiriyama A (2018). Delivery of oxytocin to the brain for the treatment of autism spectrum disorder by nasal application. Mol Pharm..

[85] Lee MR, Shnitko TA, Blue SW, Kaucher AV, Winchell AJ, Erikson DW (2020). Labeled oxytocin administered via the intranasal route reaches the brain in rhesus macaques. Nat Commun..

[86] Bowen MT (2019). Does peripherally administered oxytocin enter the brain? Compelling new evidence in a long-running debate. Pharm Res.

[87] Rault J-L (2016). Effects of positive and negative human contacts and intranasal oxytocin on cerebrospinal fluid oxytocin. Psychoneuroendocrinology..

[88] Nair AB, Jacob S (2016). A simple practice guide for dose conversion between animals and human. J Basic Clin Pharm.

[89] Kagerbauer SM, Martin J, Schuster T, Blobner M, Kochs EF, Landgraf R (2013). Plasma oxytocin and vasopressin do not predict neuropeptide concentrations in human cerebrospinal fluid. J Neuroendocrinol.

[90] Gimpl G, Fahrenholz F (2001). The oxytocin receptor system: structure, function, and regulation. Physiol Rev.

[91] Gimpl G, Reitz J, Brauer S, Trossen C. Oxytocin receptors: ligand binding, signalling and cholesterol dependence. Prog Brain Res. 2008;170;193–204. 10.1016/S0079-6123(08)00417-2 18655883

[92] Smith AL, Freeman SM, Stehouwer JS, Inoue K, Voll RJ, Young LJ (2012). Synthesis and evaluation of C-11, F-18 and I-125 small molecule radioligands for detecting oxytocin receptors. Bioorg Med Chem.

[93] Beard R, Singh N, Grundschober C, Gee AD, Tate EW (2018). High-yielding 18 F radiosynthesis of a novel oxytocin receptor tracer, a probe for nose-to-brain oxytocin uptake in vivo. Chem Commun..

[94] Vaidyanathan R, Hammock EA (2017). Oxytocin receptor dynamics in the brain across development and species. Dev Neurobiol..

[95] Neumann ID, Landgraf R (2012). Balance of brain oxytocin and vasopressin: implications for anxiety, depression, and social behaviors. Trends Neurosci.

[96] Galbusera A, De Felice A, Girardi S, Bassetto G, Maschietto M, Nishimori K (2017). Intranasal oxytocin and vasopressin modulate divergent brainwide functional substrates. Neuropsychopharmacology..

[97] Quintana DS, Westlye LT, Hope S, Nærland T, Elvsåshagen T, Dørum E (2017). Dose-dependent social-cognitive effects of intranasal oxytocin delivered with novel Breath Powered device in adults with autism spectrum disorder: a randomized placebo-controlled double-blind crossover trial. Transl Psychiatry..

[98] Guoynes CD, Simmons TC, Downing GM, Jacob S, Solomon M, Bales KL (2018). Chronic intranasal oxytocin has dose-dependent effects on central oxytocin and vasopressin systems in prairie voles (Microtus ochrogaster). Neuroscience..

[99] Bales KL, Perkeybile AM, Conley OG, Lee MH, Guoynes CD, Downing GM (2013). Chronic intranasal oxytocin causes long-term impairments in partner preference formation in male prairie voles. Biol Psychiatry..

[100] Mustoe A, Schulte NA, Taylor JH, French JA, Toews ML (2019). Leu 8 and Pro 8 oxytocin agonism differs across human, macaque, and marmoset vasopressin 1a receptors. Sci Rep..

[101] MWFT Verhees, van IJzendoorn MH, Bakermans-Kranenburg MJ, Ceulemans E, de Winter S, Santens T (2020). Combining oxytocin and cognitive bias modification training in a randomized controlled trial: effects on trust in maternal support. J Behav Ther Exp Psychiatry.

[102] Spengler FB, Schultz J, Scheele D, Essel M, Maier W, Heinrichs M (2017). Kinetics and dose dependency of intranasal oxytocin effects on amygdala reactivity. Biol Psychiatry.

[103] Lieberz J, Scheele D, Spengler FB, Matheisen T, Schneider L, Stoffel-Wagner B (2020). Kinetics of oxytocin effects on amygdala and striatal reactivity vary between women and men. Neuropsychopharmacol.

[104] Paloyelis Y, Doyle OM, Zelaya FO, Maltezos S, Williams SC, Fotopoulou A (2016). A spatiotemporal profile of in vivo cerebral blood flow changes following intranasal oxytocin in humans. Biol Psychiatry.

[105] Geng Y, Zhao W, Zhou F, Ma X, Yao S, Hurlemann R (2018). Oxytocin enhancement of emotional empathy: generalization across cultures and effects on amygdala activity. Front Neurosci..

[106] Lane A, Luminet O, Nave G, Mikolajczak M. Is there a publication bias in behavioral intranasal oxytocin research on humans? Opening the file drawer of one lab. J Neuroendocrinol. 2016;28. 10.1111/jne.12384 26991328

[107] Gelman A, Loken E (2014). Ethics and statistics: the AAA Tranche of subprime science. CHANCE.

[108] Declerck CH, Boone C, Pauwels L, Vogt B, Fehr E (2020). A registered replication study on oxytocin and trust. Nat Hum Behav.

[109] Munafò MR, Nosek BA, Bishop DV, Button KS, Chambers CD, Du Sert NP (2017). A manifesto for reproducible science. Nat Hum Behav.

[110] Poldrack RA, Baker CI, Durnez J, Gorgolewski KJ, Matthews PM, Munafò MR (2017). Scanning the horizon: towards transparent and reproducible neuroimaging research. Nat Rev Neurosci.

[111] Flannery J. fMRI Preregistration Template. 2018. https://osf.io/6juft/.

[112] Ellis CT, Baldassano C, Schapiro AC, Cai MB, Cohen JD (2020). Facilitating open-science with realistic fMRI simulation: validation and application. PeerJ..

[113] Frijling JL, van Zuiden M, Koch SBJ, Nawijn L, Veltman DJ, Olff M (2016). Effects of intranasal oxytocin on amygdala reactivity to emotional faces in recently trauma-exposed individuals. Soc Cogn Affect Neurosci.

[114] Andari E, Duhamel J-R, Zalla T, Herbrecht E, Leboyer M, Sirigu A (2010). Promoting social behavior with oxytocin in high-functioning autism spectrum disorders. PNAS..

[115] Dumais KM, Veenema AH. Presence and absence of sex differences in structure and function of the brain oxytocin system: implications for understanding the regulation of social behavior. In: Shansky RM, editor. Sex differences in the central nervous system, San Diego: Academic Press; 2016. p. 247–95.

[116] Radke S, de Bruijn ERA (2015). Does oxytocin affect mind-reading? A replication study. Psychoneuroendocrinology..

[117] Zhao W, Luo R, Sindermann C, Li J, Wei Z, Zhang Y (2020). Oxytocin modulation of self-referential processing is partly replicable and sensitive to oxytocin receptor genotype. Prog Neuropsychopharmacol Biol Psychiatry.

[118] Ludman EJ, Fullerton SM, Spangler L, Trinidad SB, Fujii MM, Jarvik GP (2010). Glad you asked: participants’ opinions of re-consent for DbGap data submission. J Empir Res Hum Res Ethics.

[119] Mello MM, Lieou V, Goodman SN (2018). Clinical trial participants’ views of the risks and benefits of data sharing. N. Engl J Med.

[120] Reiter JP (2005). Releasing multiply imputed, synthetic public use microdata: an illustration and empirical study. J R Stat Soc Ser A Stat Soc.

[121] Quintana DS (2020). A synthetic dataset primer for the biobehavioural sciences to promote reproducibility and hypothesis generation. eLife..

[122] Franco A, Malhotra N, Simonovits G (2014). Publication bias in the social sciences: unlocking the file drawer. Science..

[123] Wagenmakers E-J, Love J, Marsman M, Jamil T, Ly A, Verhagen J (2018). Bayesian inference for psychology. Part II: example applications with JASP. Psychon Bull Rev.

[124] Quintana DS, Williams DR (2017). Bayesian alternatives for common null-hypothesis significance tests in psychiatry: a non-technical guide using JASP. BMC Psychiatry.

[125] Lakens D, Scheel AM, Isager PM (2018). Equivalence testing for psychological research: a tutorial. Adv Methods Pract Psychol Sci.

[126] Quintana DS (2018). Revisiting non-significant effects of intranasal oxytocin using equivalence testing. Psychoneuroendocrinology..

[127] IJzendoorn MH van, Bakermans‐Kranenburg MJ. The role of oxytocin in parenting and as augmentative pharmacotherapy: critical issues and bold conjectures. J Neuroendocrinol. 2016;28. 10.1111/jne.12355 26709101

[128] Cho H-C, Abe S (2013). Is two-tailed testing for directional research hypotheses tests legitimate?. J Bus Res.

[129] Muthukrishna M, Henrich J (2019). A problem in theory. Nat Hum Behav.

[130] Szollosi A, Kellen D, Navarro DJ, Shiffrin R, van Rooij I, Van Zandt T (2020). Is preregistration worthwhile?. Trends Cogn Sci.

[131] Smaldino P (2019). Better methods can’t make up for mediocre theory. Nature..

[132] Wells JC, Nesse RM, Sear R, Johnstone RA, Stearns SC (2017). Evolutionary public health: introducing the concept. Lancet..

[133] Grebe NM, Gangestad SW. Oxytocin: an evolutionary framework. In: Welling LLM, Shackelford TK, editors. The Oxford Handbook of Evolutionary Psychology and Behavioral Endocrinology. New York,NY: Oxford University Press; 2019.

[134] Harari-Dahan O, Bernstein A (2014). A general approach-avoidance hypothesis of oxytocin: accounting for social and non-social effects of oxytocin. Neurosci Biobehav Rev.

[135] Meyer-Lindenberg A, Domes G, Kirsch P, Heinrichs M (2011). Oxytocin and vasopressin in the human brain: social neuropeptides for translational medicine. Nat Rev Neurosci.

[136] Luminet O, Grynberg D, Ruzette N, Mikolajczak M (2011). Personality-dependent effects of oxytocin: greater social benefits for high alexithymia scorers. Biol Psychol..

[137] Scheele D, Kendrick KM, Khouri C, Kretzer E, Schläpfer TE, Stoffel-Wagner B (2014). An oxytocin-induced facilitation of neural and emotional responses to social touch correlates inversely with autism traits. Neuropsychopharmacology..

[138] Xin F, Zhou F, Zhou X, Ma X, Geng Y, Zhao W, et al. Oxytocin modulates the intrinsic dynamics between attention-related large-scale networks. Cereb Cortex. 2018. 10.1093/cercor/bhy295. 10.1093/cercor/bhy295 30535355

[139] Eckstein M, Markett S, Kendrick KM, Ditzen B, Liu F, Hurlemann R (2017). Oxytocin differentially alters resting state functional connectivity between amygdala subregions and emotional control networks: Inverse correlation with depressive traits. NeuroImage..

[140] Ellenbogen MA, Linnen A-M, Cardoso C, Joober R (2013). Intranasal oxytocin impedes the ability to ignore task-irrelevant facial expressions of sadness in students with depressive symptoms. Psychoneuroendocrinology..

[141] Ma Y, Li S, Wang C, Liu Y, Li W, Yan X (2016). Distinct oxytocin effects on belief updating in response to desirable and undesirable feedback. Proc Natl Acad Sci.

[142] Alvares GA, Chen NTM, Balleine BW, Hickie IB, Guastella AJ (2012). Oxytocin selectively moderates negative cognitive appraisals in high trait anxious males. Psychoneuroendocrinology..

[143] Luo L, Becker B, Geng Y, Zhao Z, Gao S, Zhao W (2017). Sex-dependent neural effect of oxytocin during subliminal processing of negative emotion faces. NeuroImage..

[144] Grimm S, Pestke K, Feeser M, Aust S, Weigand A, Wang J (2014). Early life stress modulates oxytocin effects on limbic system during acute psychosocial stress. Soc Cogn Affect Neurosci.

[145] Frijling JL, van Zuiden M, Koch SB, Nawijn L, Veltman DJ, Olff M (2016). Intranasal oxytocin affects amygdala functional connectivity after trauma script-driven imagery in distressed recently trauma-exposed individuals. Neuropsychopharmacology..

[146] Horta M, Kaylor K, Feifel D, Ebner NC (2020). Chronic oxytocin administration as a tool for investigation and treatment: a cross-disciplinary systematic review. Neurosci Biobehav Rev.

[147] Hurlemann R, Patin A, Onur OA, Cohen MX, Baumgartner T, Metzler S (2010). Oxytocin enhances amygdala-dependent, socially reinforced learning and emotional empathy in humans. J Neurosci..

[148] Hu J, Qi S, Becker B, Luo L, Gao S, Gong Q (2015). Oxytocin selectively facilitates learning with social feedback and increases activity and functional connectivity in emotional memory and reward processing regions. Hum Brain Mapp.

[149] Rilling JK, DeMarco AC, Hackett PD, Thompson R, Ditzen B, Patel R (2012). Effects of intranasal oxytocin and vasopressin on cooperative behavior and associated brain activity in men. Psychoneuroendocrinology..

[150] Melby K, Gråwe RW, Aamo TO, Salvesen Ø, Spigset O (2019). Effect of intranasal oxytocin on alcohol withdrawal syndrome: a randomized placebo-controlled double-blind clinical trial. Drug Alcohol Depend.

[151] Stauffer CS, Meinzer NK, Morrison T, Wen J-H, Radanovich L, Leung D (2019). Effects of oxytocin administration on cue-induced craving in co-occurring alcohol use disorder and PTSD: a within-participant randomized clinical trial. Alcohol Clin Exp Res.

[152] Munesue T, Yokoyama S, Nakamura K, Anitha A, Yamada K, Hayashi K (2010). Two genetic variants of CD38 in subjects with autism spectrum disorder and controls. Neurosci Res.

